# “I Just Wouldn't Like Him to go Through What I Went Through as a Kid”: A Qualitative Study on the Mitigating Effects of Positive Childhood Experiences in Mothers with a History of Adverse Childhood Experiences in an Irish Population

**DOI:** 10.1007/s10597-024-01353-9

**Published:** 2024-09-14

**Authors:** Lamia Tadjine, Lorraine Swords

**Affiliations:** School of Psychology, Trinity College Dublin, College Green, Dublin 2, Ireland

**Keywords:** Adverse childhood experiences, Benevolent childhood experiences, Intergenerational trauma, Parenting, Maternal mental health, Child development

## Abstract

Adverse Childhood Experiences (ACEs) are increasingly being acknowledged as a major risk factor for instigating and sustaining cycles of trauma between mother and child. Recently, the concept of Benevolent Childhood Experiences (BCEs) has been introduced to ACEs research as a buffer against the transmission of ACEs between generations. Positive childhood experiences such as attachment to caregivers, positive peer relations and positive sense of self have been found to counteract the effects of adverse childhood experiences. The emergence of positive childhood experiences as an adaptive capacity against ACEs should be explored as a tool for psychological change, to help break the cycle of inherited trauma between generations. The present study aims to examine the lived experiences of mothers with a history of ACEs, if they consider their positive childhood experiences when parenting, and how they use these positive experiences to break the cycle of intergenerational trauma. Three women residing in a low-support service for parenting were recruited for this study. Participants were all low-income, first-time single mothers in their early thirties. A qualitative approach was designed for the study. ACEs and BCEs questionnaires were administered to participants and scores were taken into account to contextualise participant interviews. A semi-structured interview was designed in accordance with IPA guidelines. Questions were directed towards phenomenological material, focusing on participants’ understanding of their experiences as mothers. Analysis of the interview data revealed three superordinate themes (replicating positive experiences, creating new positive experiences and protecting children from intergenerational trauma) related to participants' BCEs, their children’s BCEs and their desire to break the cycle of intergenerational trauma. The findings of this study, namely that participants intentionally tried to create positive experiences with their own children through drawing on their own positive experiences in childhood, supports the idea that BCEs are a legitimate source of adaptive capacity for mothers with ACEs. Parenting interventions for parents with ACEs should be developed taking into account ACE and BCE scores.

## Introduction

Adverse childhood experiences (ACEs) are defined as traumatic events experienced during childhood that affect the health and functioning of a person throughout their lifetime (Felitti et al., 1998). ACEs include three core categories within which childhood adversity is defined. Family dysfunction, abuse and neglect broadly define ACEs, however individual ACEs within these categories are incredibly diverse in their nature and impact (Boullier & Blair, 2018). Children who are exposed to ACEs are at greater risk of a variety of different physical and psychological problems in adulthood, and many studies have established and emphasised these risks and their effects on the individual (Young-Wolff et al., 2021; Narayan et al., 2021; Cuijpers et al., 2011; Feletti et al., 1998). Exposure to stress in infancy has been shown to cause permanent damage to the developing brain, causing social, emotional and cognitive impairment. This often leads to the development of behaviours that are harmful for physical and mental health later in life (Boullier & Blair, 2018). Childhood adversities have been found to be significantly associated with medical issues such as coronary heart disease, diabetes and increased sexually transmitted diseases; and psychological issues such as mood disorders, substance misuse and increased suicide attempts (Cuijpers et al., 2011).

The impact of ACEs on an individual’s life is sequenced and often begins in utero (Racine et al., 2018). As pregnancy is a physical and psychological transformative period, the experience of pregnancy itself often triggers the development of issues in mothers with a history of ACEs which subsequently affect their child throughout the pregnancy period and thereafter. Mothers begin to reflect on their own childhood as the prospect of becoming a parent becomes tangible, and traumatic childhood experiences resurface (Narayan et al., 2017). During the pregnancy period women who have experienced trauma in childhood—particularly those who were exposed to abuse—are more likely to smoke, manifest preeclampsia or have a pregnancy length shorter than 37 weeks (Roberts et al., 2013).The influence of maternal ACEs on the child continue throughout the maternal experience, and extend beyond the direct physical impact that occurs in pregnancy. The emotional and psychological issues associated with a history of childhood adversity often undermine parenting capacity, and associations have been found between ACEs and lower parenting competence, greater parenting stress and aggressive conflict management (Bailey et al., 2012). The perpetration of dysfunctional parenting as a result of maternal ACEs manifests in a variety of different ways, often depending on the type of ACEs experienced. For example, mothers who have experienced emotional neglect and abuse in childhood often express impatience and frustration during interactions with their child, while mothers with a history of sexual abuse tend to emotionally withdraw from interactions with their child (Bailey et al., 2012). Research in this area has primarily focused on the replication of dysfunctional parenting carried out by parents with a history of childhood trauma showing the long term impact, and more importantly the transmission of trauma between generations. Recently, research on ACEs has shifted from describing the long-term impact of ACEs, to preventing ACEs in children; most notably in families with a history of maternal ACEs (Narayan et al., 2021).

Recent research on ACEs has identified positive childhood experiences as a potential source for promoting psychological change and resilience in mothers with a history of childhood trauma (Davis & Narayan, 2020; Narayan et al., 2021). Positive childhood experiences or benevolent childhood experiences (BCEs) are early favourable experiences that elicit resilience in the individual. BCEs encapsulate feelings of safety, support and attachment throughout childhood. Conceptually, they exist as a counterpart to ACEs and have been established as a viable source for positive growth for those with a history of childhood trauma (Davis & Narayan, 2020; Narayan et al., 2021). While ACEs and BCEs often co-occur in childhood, ACEs research has shown that even within high risk cases, positive childhood experiences drastically counteract the negative effects of maltreatment in childhood. For example, among maltreated children, secure attachment to extended family and good peer relationships are associated with greater resilience and lower levels of psychopathology in adulthood (Collishaw et al., 2007; Narayan et al., 2018). Resilience research has defined positive childhood experiences as promotive factors that directly reduce the risk of unfavourable outcomes in both low and high risk individuals (Narayan et al., 2018). Promotive factors are defined as positive contextual, social and individual factors that encourage positive development from birth. For example, promotive factors are often external resources such as good familial support and socio-economic status (Zimmerman, 2013).

Research examining the mitigating effects of BCEs in pregnant women has also been promising. As mentioned, during the pregnancy period childhood memories become more salient as mothers contemplate their experiences and consider how they would like to raise their child (Davis & Narayan, 2020). Research on BCEs has found that those with a history of positive childhood experiences have fewer mental health problems in pregnancy, better prenatal health and better child outcomes, as they are able to harness resilience from their positive experiences and draw upon them throughout their pregnancy and thereafter (Davis & Narayan, 2020). Moreover, as mothers with a history of BCEs have fewer physical and mental health problems, the transmission of child maltreatment through parenting is mitigated greatly, revealing an intergenerational element to BCEs (Narayan et al., 2019). Given the nature of ACEs, they often co-occur throughout childhood which increases their negative impact on the individual (Liming, 2019). However, research on BCEs has found that even in mothers with four or more ACEs, harnessing the positive impact of benevolent childhood experiences can encourage more resilient functioning (Crouch et al., 2019). Findings from studies examining positive childhood experiences found that mothers with prominent benevolent childhood experiences were more conscious of mitigating their children's exposure to traumatic events (Naryan et al., 2019). Positive childhood experiences facilitate resilience in parents, allowing them to protect their own children from projections of their adverse experiences. The nature of these internal positive experiences as potential tools for resilience means that mothers can recreate positive caregiving moments from their experiences without having to rely on any external factors for support (Naryan et al., 2019).

Given that previous research has suggested an adaptive function to drawing on BCEs throughout motherhood, there is good reason to explore this further especially emphasising the intergenerational nature of BCEs from mother to child. Parenting styles and methods have long been recognised as behaviours that are shared and replicated between generations, with parents engaging in similar parenting practices that they experienced growing up (Belsky et al., 2009). However, research in this area has primarily focused on the replication of dysfunctional and abusive parenting carried out by parents with a history of childhood trauma, while little attention has been given to the positive experiences that parents with a history of ACEs attempt to pass on to their children (Davis & Narayan, 2020; Narayan et al., 2019).

## The Present Study

The emergence of BCEs as protective factors presents an interesting line of inquiry for investigating the influence that positive childhood experiences have on the transmission of ACEs between generations. As this is an emerging subject of interest within the realm of ACEs research, in-depth, qualitative literature examining the specific experience of how mothers with ACEs use their BCEs as a source of resilience is sparse. The purpose of the present study is to explore the lived experiences of mothers with a history of ACEs, if they consider their positive childhood experiences when parenting their child, and how they use these positive experiences to break the cycle of intergenerational trauma. Examining how mothers with a history of ACEs create BCEs with their children could contribute to future mental health and parenting interventions for mothers with a history of trauma. BCEs could be a potential internal source of resilience for mothers with ACEs giving them a sense of control over their trauma, subsequently helping to break the cycle of trauma between generations.

### Research Design

As this study is interested in the specific experience of mothering with a history of childhood trauma, a qualitative approach was deemed most appropriate to extensively explore this topic. Interpretative phenomenological analysis (IPA) was chosen as the method of analysis. IPA attempts to explore how participants make sense of their lived experiences, and examines these experiences through the subjective perception of the participant with the aim of gaining a rich understanding into the participant’s inner world (Smith et al., 2009). The Adverse Childhood Experiences questionnaire and the Benevolent Childhood Experiences questionnaire were used to contextualise and understand the scope of trauma experienced by participants.

### Participants

Three women residing in a low support service for parenting were recruited for this study. The service provides practical support and preparation for independent living for lone parents through parenting workshops and interventions. Lone parents are referred to the service by the government’s child protection and social work department. Seven potential participants (capacity of service) were initially mailed information sheets about the study and were contacted after a week to gauge their interest in participating in the study. Four mothers responded to indicate their willingness to get involved, but one mother subsequently chose to withdraw. The resulting sample of three was deemed appropriate as IPA requires an in-depth exploration of participants' experiences, therefore smaller samples contribute to more intentional and rich analysis (Smith et al., 2009). Participants were all low-income, first-time single mothers in their early thirties.

### Materials

The Adverse Childhood Experiences (ACEs) scale is a ten-item scale used to measure childhood trauma (Felitti et al., 1998). The scale addresses experiences of family dysfunction, abuse and neglect in childhood, such as incarcerated relatives, sexual abuse and physical neglect. For each event listed, respondents are asked to indicate if they had experienced it or not during the first 18 years of their life. The number of events experienced are tallied and respondents’ scores can range from 0 to 10.

The Benevolent Childhood Experiences (BCEs) scale was also given to participants. The BCEs scale is a ten-item scale used to assess positive early-life experiences in adults with a history of ACEs. The scale primarily addresses feelings of safety, attachment and stability, such as having an adult with whom you felt safe, having a consistent caregiver and a predictable home routine (Narayan et al., 2018). For each event listed, respondents are asked if they had experienced it or not during the first 18 years of their life. The number of events experienced are tallied and respondents’ scores can range from 0 to 10. Both scales were used to contextualise interview material.

A semi-structured interview was designed in accordance with IPA guidelines, as outlined by Smith et al. (2009). Questions were directed towards phenomenological material, focusing on participants’ understanding of their experiences as mothers. Questions were broad and open ended and allowed participants to express their unique experiences of parenting with their ACEs and BCEs (Smith et al., 2009). The interview questions were as follows:

1. Can you describe a happy or positive memory from your childhood?
2. What are the main differences between your childhood and your child’s childhood?
3. Can you describe a typical day with your child?
4. What childhood experiences influence your parenting the most?
5. How do you feel about being a mother?
6. Is motherhood what you expected it to be?

### Procedure

Ethical approval was granted by the Research Ethics Committee of the School of Psychology at Trinity College Dublin, and within the parenting service itself. Individual interviews with each participant took place in the parenting service. Before interviewing began, informed consent was obtained from participants. They were given the Adverse Childhood Experiences (ACEs) scale and the Benevolent Childhood Experiences (BCEs) scale to complete and report how many experiences were applicable to them in each scale. This was done to confirm and contextualise participants’ experiences of ACEs and BCEs, while the subsequent interview aspect of the study explored the influence of BCEs in mitigating the transmission of their ACEs through their parenting. Interviews lasted between 30 and 40 min. Participants were recorded, and their interviews were transcribed verbatim using NVivo 12. Experiences relevant to the study were probed where appropriate, as they were revealed when participants answered the listed questions above.

### Analysis

ACEs and BCEs scores were tallied up for each participant. Interview data was analysed using the guidelines recommended for IPA by Smith et al. (2009). Pseudonyms were used to protect participants’ identities. Transcripts were read thoroughly, and then re-read while listening to the interview audio. This allowed for an immersive experience that ensured participants were better understood, allowing for a more comprehensive analysis of the data. Transcripts were individually analysed for emergent themes, and any information relevant to the research question was noted. Themes were drawn from each individual case, and connections between these themes were made where relevant. ACE and BCE scores were considered throughout the analysis process to further understand the extent of the participants’ trauma.

## Results

Participants’ ACE and BCE scores are detailed in Table 1 (attached). The original ACE study found that of 17,337 respondents, only 12.5% had experienced four or more ACEs (Boullier & Blair, 2018). ACE scores within this sample ranged from four to seven, indicating early adversity across all three participants. With regards to BCE scores, the original study found that in a sample of 101 pregnant women, the average BCE score was seven. Participants in this study reported only experiencing between one and six, and two out of the three participants had an ACE score that exceeded their BCE score. Analysis of the interview data revealed three superordinate themes related to participants' BCEs, their children’s BCEs and their desire to break the cycle of intergenerational trauma. Participants spoke about intentionally replicating their own positive childhood experiences with their children, prioritising their children’s interests and feelings in creating positive memories that are unique to their relationship, and protecting their children from intergenerational trauma through reflecting on both their positive and negative experiences in childhood.

**Table 1:** Participant ACE and BCE scores

Participant	Number of ACEs	Number of BCEs
Lauren	4	5
Alison	7	6
Nicola	6	1

### Replicating Positive Experiences

Both Lauren and Alison expressed that they make a conscious effort to replicate the positive aspects of their childhood with their children now. While their circumstances in terms of wealth and space are significantly worse than what they experienced in childhood, Lauren and Alison try to reproduce their positive childhood experiences as best as they can:

We do a lot of messy play. Because I was always out in the back garden and we don’t have a garden, but I was always out making mud pies and everything like that. But I filled [a drawer] with shaving foam and [cosmetics] and she’s very happy to just pour loads of stuff together and make potions and things like that. (Lauren)

Alison’s positive experiences were centred around the closeness she had with her mother and siblings. She shared that her relationship with her father was always strained as he physically abused her, but the emotional closeness she had with her mother and siblings was a positive aspect of her childhood. Although she is a lone parent with no family close by, Alison explained that she tries to replicate the experiences that helped her feel close to her mother and siblings with her child now:

The positive aspects were my mum, whenever she had the time to be with us it was one of the best days. I just imagine the days we would get to sleep with her in her room when our Dad was travelling. All of us would just come, she had a very big bed. So I just feel like each time me and [child’s name] are also together it’s like that. I say come to mommy’s bed. We used to get so much joy out of those kinds of things. (Alison)

### Creating New Positive Experiences

Participants shared a strong desire to ensure that their relationships with their children were open and focused on creating positive experiences. Participants emphasised allowing their children to take the lead when spending time together, so that activities are centred around their own wants and needs. For example, when Nicola was asked to describe a typical day with her child, she said:

I'd say like, mischievous, funny, like, lots of adventure, because he's a very curious child and he's very hyper, so it's an active day as well…we always make sure we're doing something and that he's happy and I can look back and show him and say to him, ah you had such great fun on this day (Nicola).

Alison and Lauren also expressed that creating positive experiences with their children involves prioritising their children’s interests. Alison finds that her child is his happiest when he’s outside, and so she tries to take him out as much as she can:

When he’s not in school we’ll go out…as long as he gets on the Luas that day or we go for a long walk he’s very happy. The days that we spend at least five hours outside the house, he's the happiest person. I get tired but he's still happy in the buggy, you know? (Alison)

Lauren shared that she prioritises her daughters happiness by giving her choices. In doing this, Lauren has found that her daughter has become more confident and self-assured:

I give her choices you know that way… I signed her up to join a choir and she’s like, I don't want to join the choir I want to be a rock star and I'm like, OK, you know…I really, really promote her autonomy and her knowing herself (Lauren)

Another facet of creating positive experiences for Nicola is including experiences that she did not experience as a child. Nicola explained that a lot of positive feelings and experiences were lacking in her childhood and wants to make sure that her child feels fulfilled and happy through the time that they spend together:

It feels good that I'm able to give him something that I wasn't able to have. Like he'd be a lot happier [than I was as a child]…because of summer holidays were sort of making fun out of, like little things. Like getting him sand and water, more sensory things, what I wouldn’t have done as a kid. Like more outdoor things that I wouldn't have done as a kid either…but it's more about making little memories out of little things.

Lauren expressed similar feelings about her upbringing, and shared that she wants to make sure that her relationship with her daughter is founded on positive feelings and experiences, as she does not want her daughter to experience the negative emotions she felt during her childhood. When asked whether her experiences as a mother were what she expected, Lauren said that she found motherhood very rewarding through the positive moments she has with her child:

I guess we have a really good connection, though. Like, I feel like she has that emotional support that I didn't have…it was very critical and controlling kind of environment that I was brought up in…I don't want her to feel like I felt growing up, which was completely invalidated. [Motherhood] is very rewarding. I love being with her, you know, we’re great company and we have a great laugh together. (Lauren).

### Protecting Children from Intergenerational Trauma

A common thread tying participants' experiences together was the insight that they had on their childhood trauma. This was mostly evident with Alison’s experience of parenting, and she explained trying replicate the good parenting she experienced from her mother:

What I learned from my mother is she single handedly - although she had a husband - she single handedly made sure her kids don’t lack anything. She was very industrious…the thing I learned from her is she was really focused on her children and the children's wellbeing. She wanted the best schools and a nice house [for us]. (Alison)

Alison also shared that reflecting on the trauma she experienced from her father helped her gain insight into the type of parent she *did not* want to be. She made it very clear that the parenting she experienced from her father was not going to be passed on to her child now. This realisation also influenced her to leave her abusive ex-partner:

My dad was really a big beater, he liked to beat a lot, you know? So when I think of what he used to do to me, like, it really makes me be very careful with the way I treat my baby because I feel it can affect them…[beating] was very normal, a baby could spill that water there now, and they would beat the baby for spilling the water. They feel the baby needs to be intelligent… I want to protect him from fighting, like I experienced my mum and Dad fighting a lot. Like every day. The environment was always tense…I don't think I would like [my child] to live in that kind of [environment]. I’d just prefer to raise him alone. (Alison)

Lauren and Nicola expressed that their insight was solely brought on by their experiences of bad parenting. Lauren spoke about the control she experienced from her parents and Nicola shared that the biggest influence to her parenting was her mums addiction and the trauma she endured from being exposed to it:

There would have been a lot of control, not the kind of violent raging control, more covert. So it was very, very hard to figure it out. I took a lot of therapy later in life to figure out what was actually going on…I’m part of that dysfunctional system and I have a responsibility to myself and my daughter to, you know, keep working on it…like [my childhood] was like overly controlled and authoritative, and I suppose I can be a bit permissive. (Lauren)

I don’t drink. I won’t take any prescriptions I could get addicted to because there is that addictive gene in my family, and I just wouldn't like him to go through what I went through as a kid. So I stay away from alcohol and stuff like that…When I went to a new doctor, the first thing I told him when we were having, like our consult. I don’t want him to prescribe things that are addictive, like anything that has benzos or stuff because the addictive gene is there. (Nicola)

## Discussion

The purpose of the present study is to explore the lived experiences of mothers with a history of ACEs, if they consider their positive childhood experiences when parenting their child, and how they use these positive experiences to break the cycle of intergenerational trauma. Three themes emerged: 1. Replicating positive experiences, 2. Creating new positive experiences and 3. Protecting children from intergenerational trauma.

While parenting research over the years has emphasised the replication of parenting practices between generations, the themes that emerged in this study, coupled with ACE and BCE scores, showed that participants in this study expressed a strong desire to protect their children from the trauma that they had endured in childhood. These findings offer an alternative perspective to the parenting expected of mothers with childhood trauma.

**Replicating Positive Experiences:** The first theme showed that maternal BCEs did in fact help to mitigate the intergenerational transmission of trauma, as participants shared that they do make an effort to pass on their BCEs to their children. Mothers in the study shared that they actively try to replicate their positive experiences with their children. This finding is in line with previous findings about the restorative and intergenerational nature of BCEs, where BCEs can counteract the effects or influence that ACEs have on an individual (Crandall et al., 2019; Narayan et al., 2021). Once ACE and BCE scores were considered with reference to participant transcripts, it was clear that the number of BCEs directly influenced participants’ ability to diminish the impact that ACEs had on their day-to-day life and their parenting. For example, Nicola had only one BCE and six ACEs, and expressed that she did not have any positive experiences that she draws on when mothering her child now. Despite this, Nicola expressed a strong desire to protect her child, and explained that her insight was rooted in her own experiences of maltreatment. While this insight is interesting given that a plethora of research exists to explain the replication of parenting practices between generations, studies have found that some maltreated mothers do break the cycle of intergenerational trauma by harnessing their negative experiences as motivation. For example, a study carried out with mothers who grew up in care found that participants' mothering was driven by a desire to parent differently to how they were parented, such that their children did not end up in care as well (Aparicio, 2016). It is also important to note that while maternal ACEs predict a higher probability of poorer outcomes for both mother and child, risk factors like ACEs do not directly determine the outcome of any individual (Narayan et al., 2021).

**Creating New Positive Experiences:** Participants also expressed that they made an effort to create positive childhood experiences with their children that are unique to their relationship. While participants recalled these positive memories, some of their anecdotes were grounded in memories of maltreatment that they experienced in childhood. For example, when Nicola and Lauren spoke about the positive experiences they have with their children, they also spoke about the aspects of their childhood that negatively affected them. These negative intrusions are often common for those that have experienced trauma, and research on mothers with ACEs and BCEs has outlined the importance of considering the intersections between positive and negative memories (Narayan et al., 2017). When researching positive childhood experiences in mothers with ACEs, Narayan et al. found that the degree to which participants spontaneously spoke about their experiences of maltreatment was associated with higher levels of total maltreatment. Within the context of this study, both Nicola and Lauren had experienced a great amount of ACEs (≥ 4). Considering this, a relevant concept to take into account when trying to understand participants' ability to create positive experiences in spite of their ACEs is resilience. Resilience is understood as the ability to adjust or “bounce back” after having negative experiences (Southwick et al., 2014). While some research suggests that resilience is fostered through healthy environments, aspects of resilience are predicated on innate individual differences. ACEs research has shown that resilience diffuses the negative impact ACEs have on an individual in adulthood, which provides a possible understanding for the insight shown by participants. Recently, studies have shown the moderating effects of resilience in adults with a history of ACEs, providing insight into the participants ability to persevere and attempt to break the cycle of trauma.

**Protecting children from intergenerational trauma:** All participants in this study spoke about breaking the cycle of trauma in their respective families. Participants described a desire to protect their children from the maltreatment that they experienced, and shared that this was the greatest influencing factor to their parenting. Participants also shared that they were very cognisant of the intergenerational aspect of trauma and maltreatment. For example Lauren shared that she has learned about this through going to therapy and acknowledges that it is something she has to constantly work on, and Nicola shared her insight on the genetic aspects of addiction in her family. This finding is similar to findings from recent studies about parenting with ACEs. For example, Woods-Jaeger et al. (2018) found that a common response to ACEs from their participants was an aspiration for their children to have better lives than they themselves had.

Strengths and Limitations: Although IPA allows for small sample sizes, the study would have benefitted from having a larger sample size so that the influence of mothering with ACEs and BCEs could be better understood. Further studies should expand on participant demographics and interview single fathers, or couples of varying ages and ethnicities with a history of ACEs. The study would have also benefited from conducting multiple interviews with participants to enhance the richness of the data. Finally, this study presents personal accounts of parenting from a niche sample, meaning that the findings of this study cannot be applied to the general population. Although experiences of mothering with a history of ACEs and BCEs is nuanced, understanding how BCEs are drawn upon by mothers with ACEs can inform parenting interventions to help prevent the intergenerational transmission of trauma through parenting. The study itself, though limited in size, provides a foundational piece of research within an Irish context about the influence of BCEs in parenting and the need for individualised parenting support interventions considering ACEs and BCEs.

Conclusion: The findings of this study, namely that participants intentionally tried to recreate and create positive experiences with their own children, supports the idea that BCEs are a legitimate source of adaptive capacity for mothers with ACEs. As previously mentioned, the nature of these internal positive experiences as potential tools for resilience means that mothers can recreate positive caregiving moments from their experiences without having to rely on any external factors for support. This finding can be used to help prevent the transmission of ACEs between generations within assessment and screening contexts for mothers. For example where BCEs exist in mothers, a strength based approach should be taken to harness the adaptive capacity of their BCEs, and in the instance of low BCE scores, more parental support and education can be offered to the individual. This study is also the first qualitative study to examine BCEs in an Irish population. The qualitative nature, and method of analysis used in this study allowed for an in-depth exploration of the experiences of an understudied population within an Irish context.

## Data Availability

Due to the sensitive nature of the questions asked in this study, survey respondents were assured raw data would remain confidential and would not be shared.

## Appendix

### Adverse Childhood Experiences (ACEs) Questionnaire

Adverse Childhood Experience (ACE) Questionnaire Finding your ACE Score.

While you were growing up, during your first 18 years of life:

### Benevolent Childhood Experiences (BCEs) Questionnaire

When you were growing up, during your first 18 years of life, did you have:

**Table:**

Item	Yes	No
1. At least one caregiver with whom you felt safe?		
2. At least one good friend		
3. Beliefs that gave you comfort		
4. Enjoyment at school		
5. At least one teacher that cared		
6. Good neighbours		
7. An adult (not a parent/ caregiver or the person from *1) who could provide you with support or advice		
8. Opportunities to have a good time		
9. Like yourself or feel comfortable with yourself		
10. Predictable home routine, like regular meals and a regular bedtime		
Total Yes’s = BCE Score		
